# Supplementary material for: 3D printing direct to industrial roll-to-roll casting for fast prototyping of scalable microfluidic systems
Source: PLoS One. 2020 Dec 28;15(12):e0244324. doi: 10.1371/journal.pone.0244324 (PMC7769481; doi:10.1371/journal.pone.0244324)
Supplement: S1 File — (PDF) [file pone.0244324.s001.pdf]

Alicona Imaging GmbH
Dr.-Auner Strasse 21a
A-8074 Raaba/Graz

# Measurement Report

# 3D Viewer

## Profilometry Baton 2 Sample 1A 10-8-2019 AsSc Crop

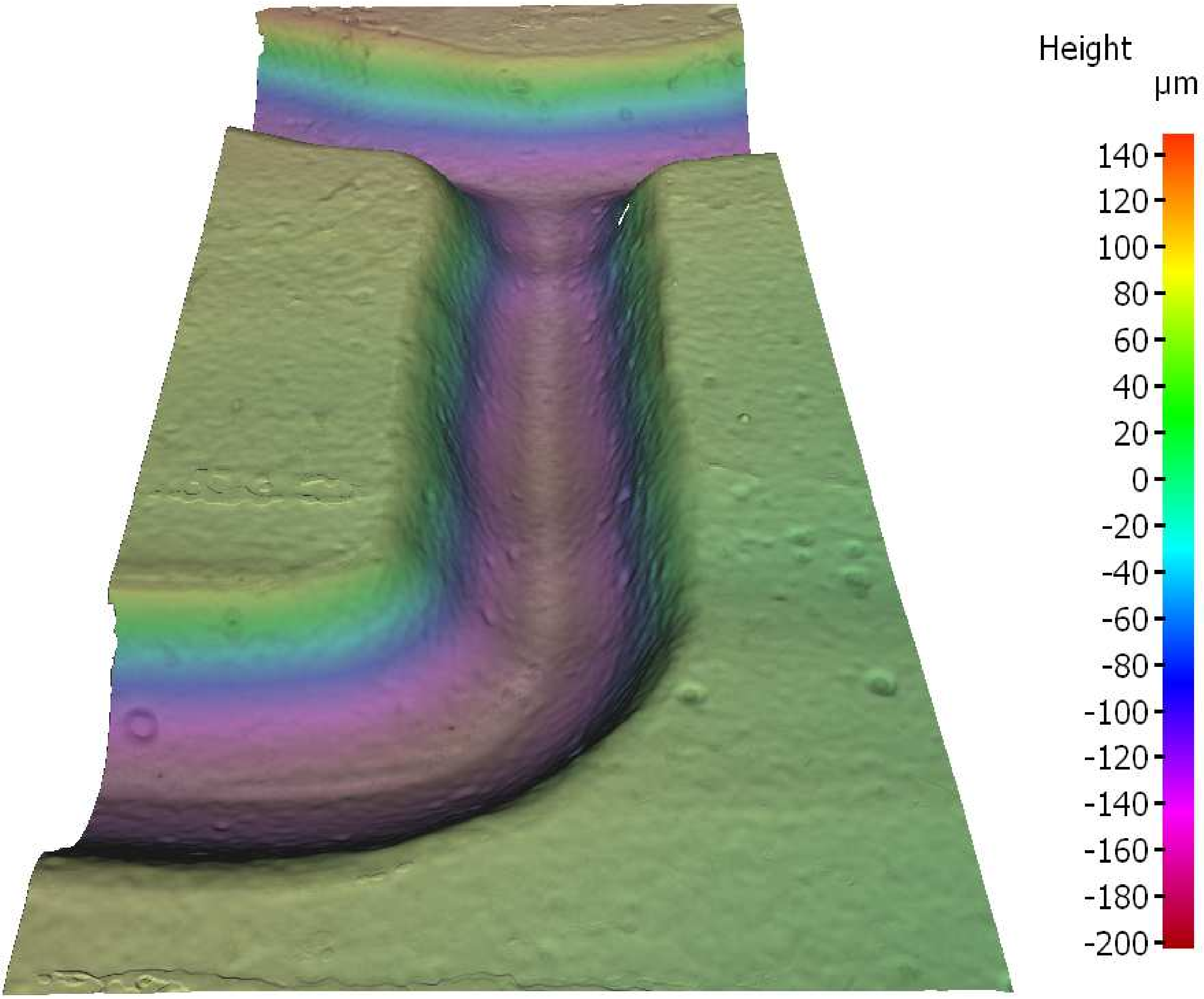

Alicona Imaging GmbH
Dr.-Auner Strasse 21a
A-8074 Raaba/Graz

# Measurement Report

# ProfileFormMeasurement

## Measurement

Profilometry Baton 2 Sample 1A 10-8-2019 AsSc Crop

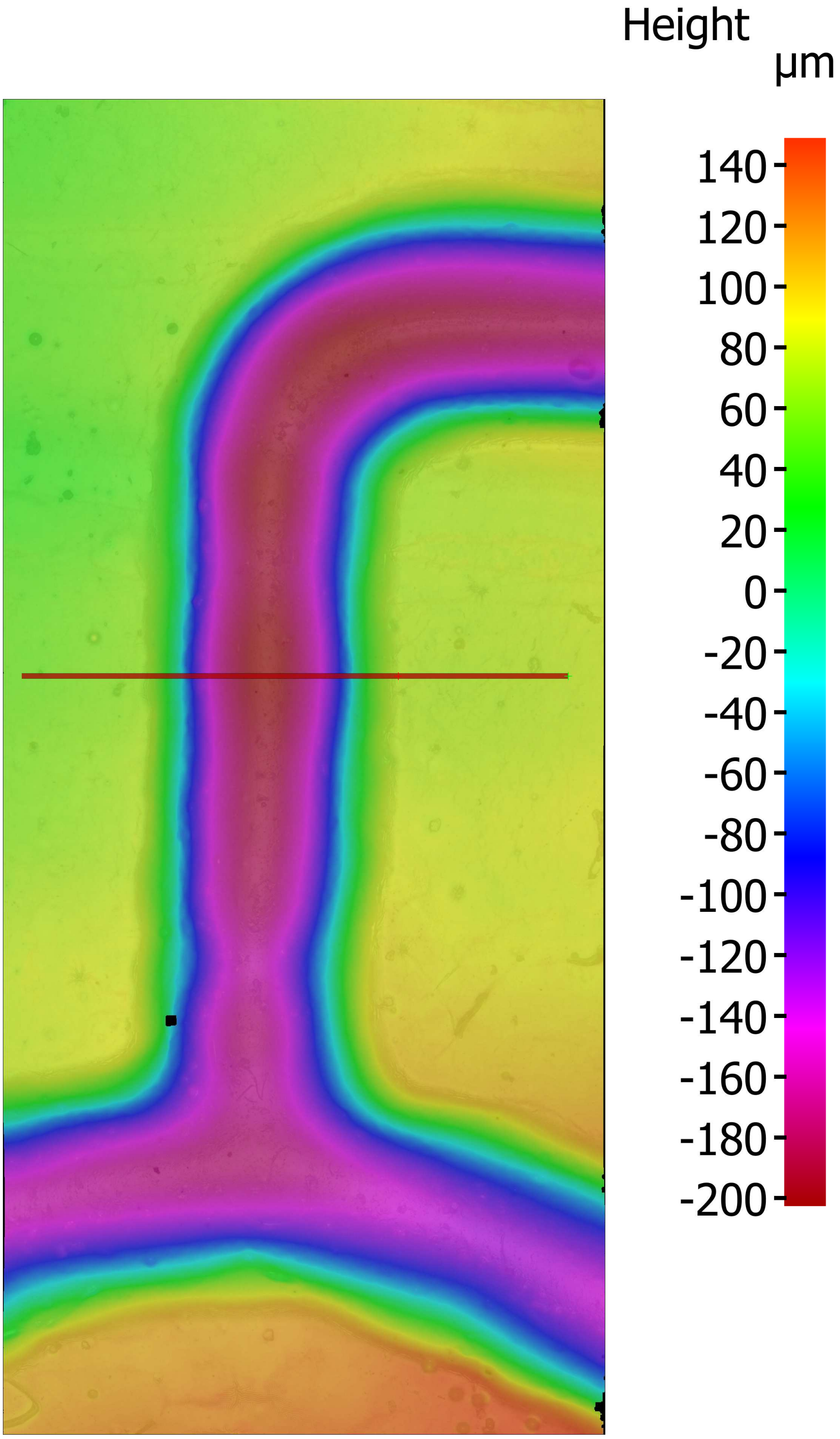

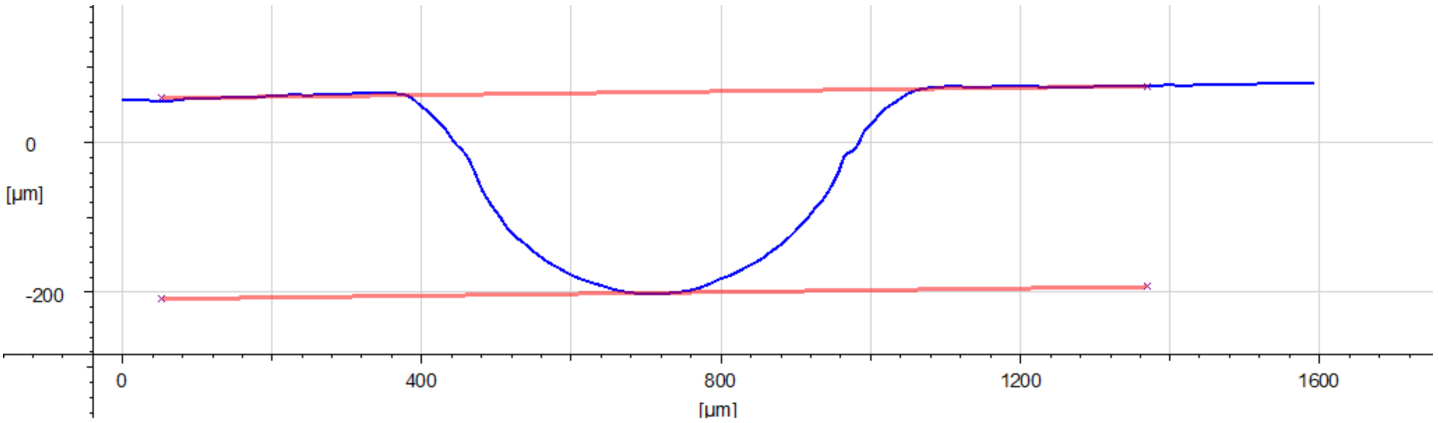


| | | |
|---|---|---|
| ReferencePosition | l: 1.5918mm | z: 79.9266µm |
| MeasurePosition | l: 0.0000nm | z: 57.8410µm |
| RelativeMeasurement | Δl: -1.5918mm | Δz: -22.0856µm |
| | Angle: 180.7949° | Distance: 1.5920mm |
| Filter: | No Filter - Primary profile | |

# Heightstep

| | Angle [°] | Distance [µm] | P1.x [µm] | P1.y [µm] | P2.x [mm] | P2.y [µm] | P3.x [µm] | P3.y [µm] | P4.x [mm] | P4.y [µm] |
|---|---|---|---|---|---|---|---|---|---|---|
| Heightstep 1 | 0.7081 | 268.8921 | 51.6074 | 59.5530 | 1.3700 | 75.8464 | 51.6074 | -209.3596 | 1.3700 | -193.0663 |

Alicona Imaging GmbH
Dr.-Auner Strasse 21a
A-8074 Raaba/Graz

alicona

# Measurement Report

## Profilometry Baton 2 Sample 1A 10-8-2019 AsSc Crop

Metric size: 1.7546mm x 3.8910mm
Size: 1595 x 3537 points
2019-10-08T13:32:46

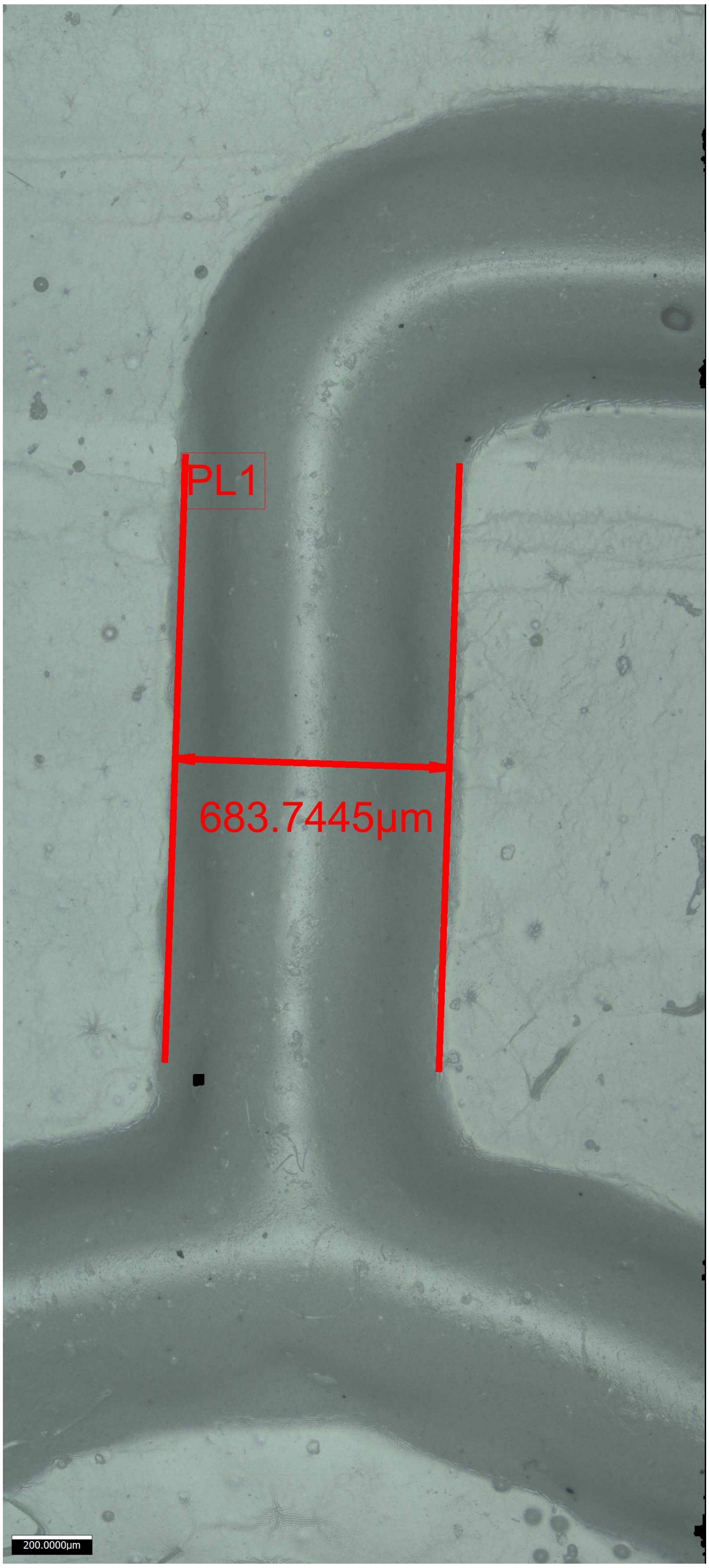

Alicona Imaging GmbH
Dr.-Auner Strasse 21a
A-8074 Raaba/Graz

# Measurement Report

# ProfileFormMeasurement

## Measurement

Profilometry Baton 2 Sample 1A 10-8-2019 AsSc Crop

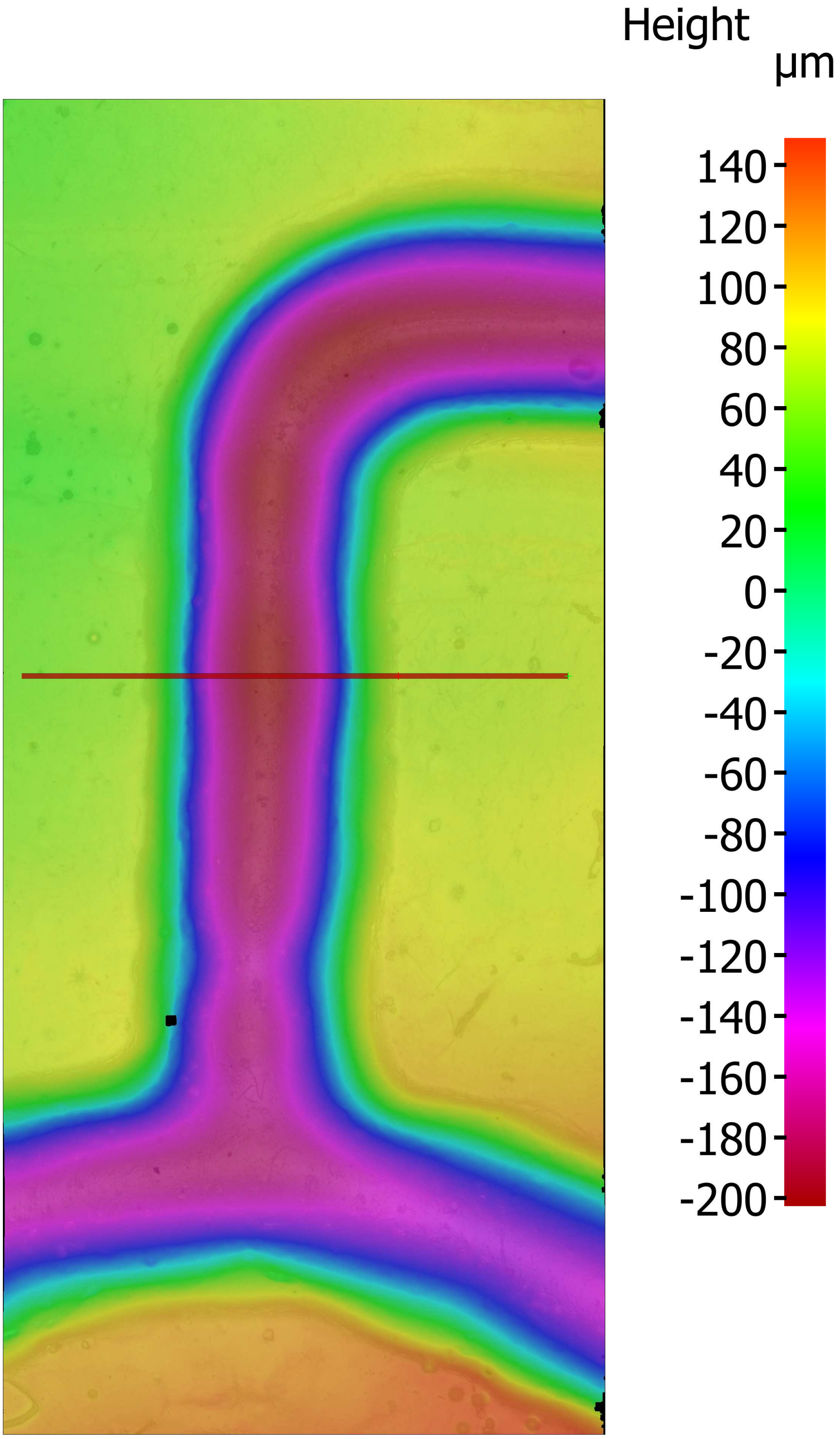

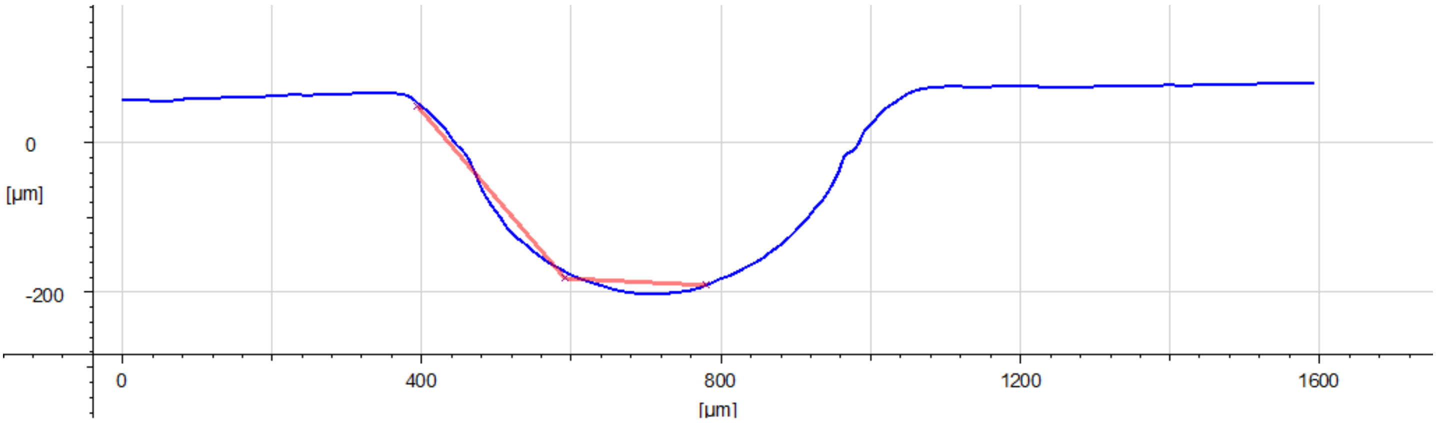


| | | |
|---|---|---|
| ReferencePosition | l: 1.5918mm | z: 79.9266µm |
| MeasurePosition | l: 0.0000nm | z: 57.8410µm |
| RelativeMeasurement | Δl: -1.5918mm | Δz: -22.0856µm |
| | Angle: 180.7949° | Distance: 1.5920mm |
| Filter: | No Filter - Primary profile | |

## Angle

| | Angle [°] | Apex X [µm] | Apex Y [µm] |
|---|---|---|---|
| Angle 1 | 133.3218 | 590.9938 | -181.7606 |
