# Supplementary material for: 3D printing direct to industrial roll-to-roll casting for fast prototyping of scalable microfluidic systems
Source: PLoS One. 2020 Dec 28;15(12):e0244324. doi: 10.1371/journal.pone.0244324 (PMC7769481; doi:10.1371/journal.pone.0244324)
Supplement: S2 File — (PDF) [file pone.0244324.s002.pdf]

Alicona Imaging GmbH
Dr.-Auner Strasse 21a
A-8074 Raaba/Graz

Measurement Report

3D Viewer

Profilometry Baton 2 Sample 2A 10-8-2019 AsSc Crop

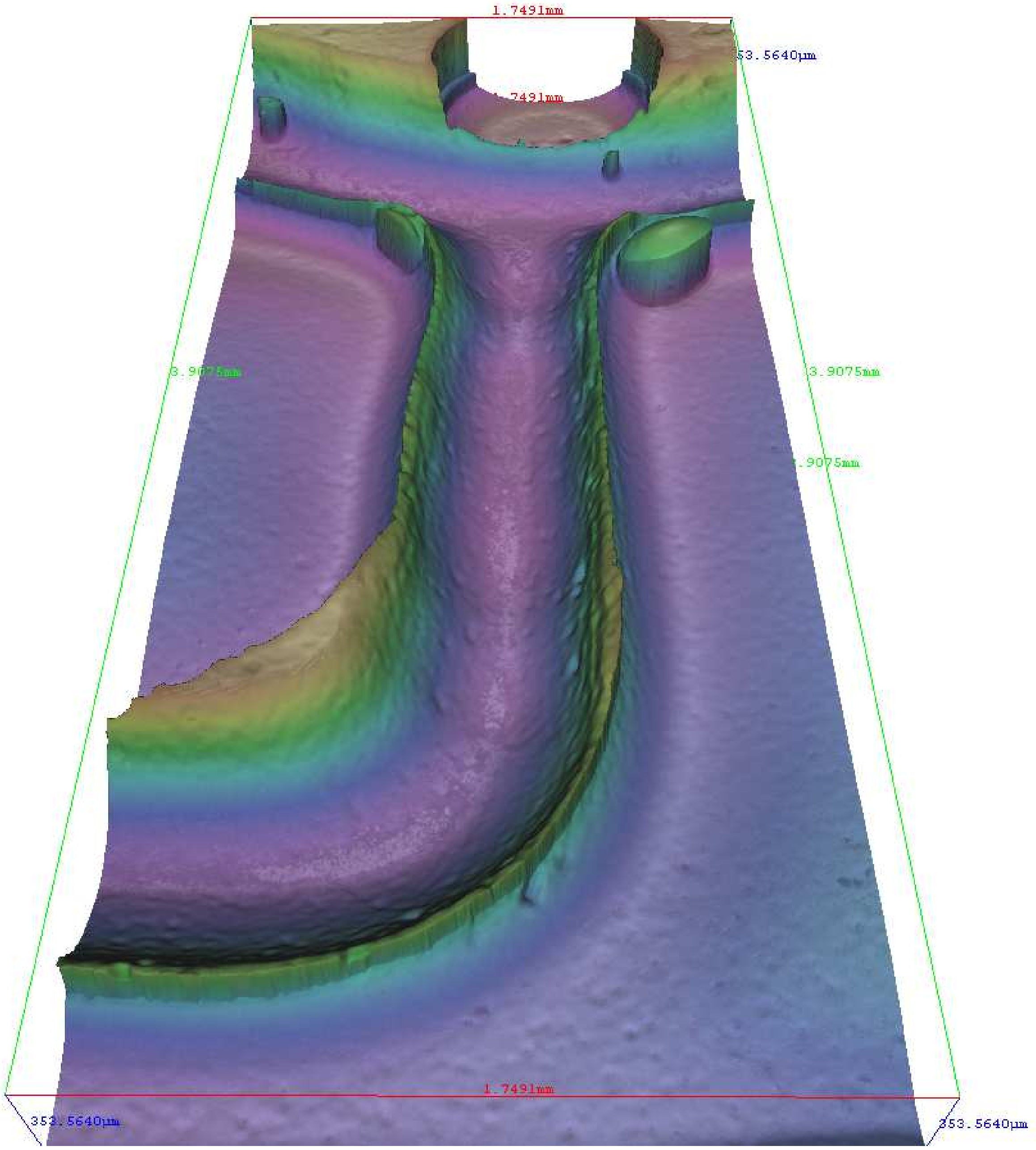

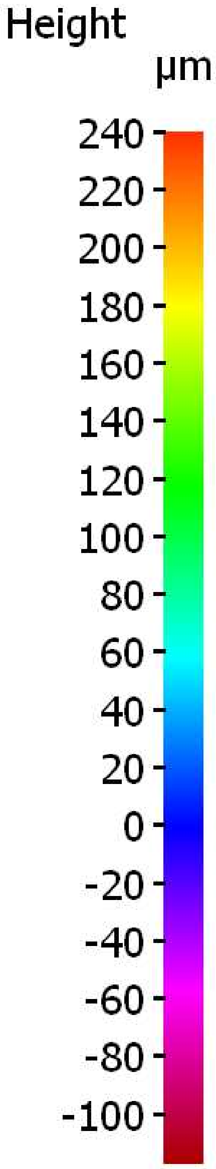

Alicona Imaging GmbH
Dr.-Auner Strasse 21a
A-8074 Raaba/Graz

alicona

Measurement Report

ProfileFormMeasurement

Measurement

Profilometry Baton 2 Sample 2A 10-8-2019 AsSc Crop

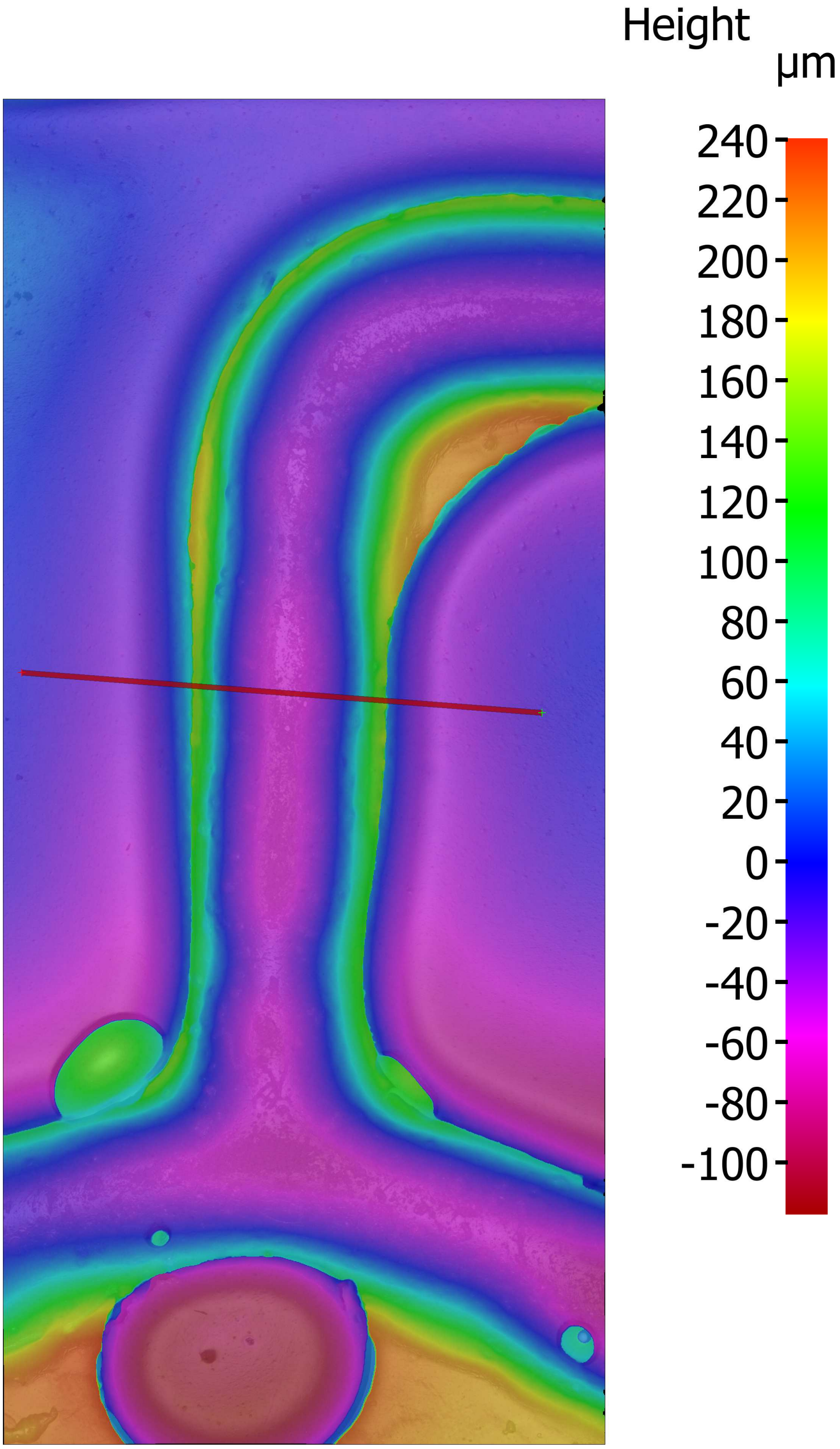

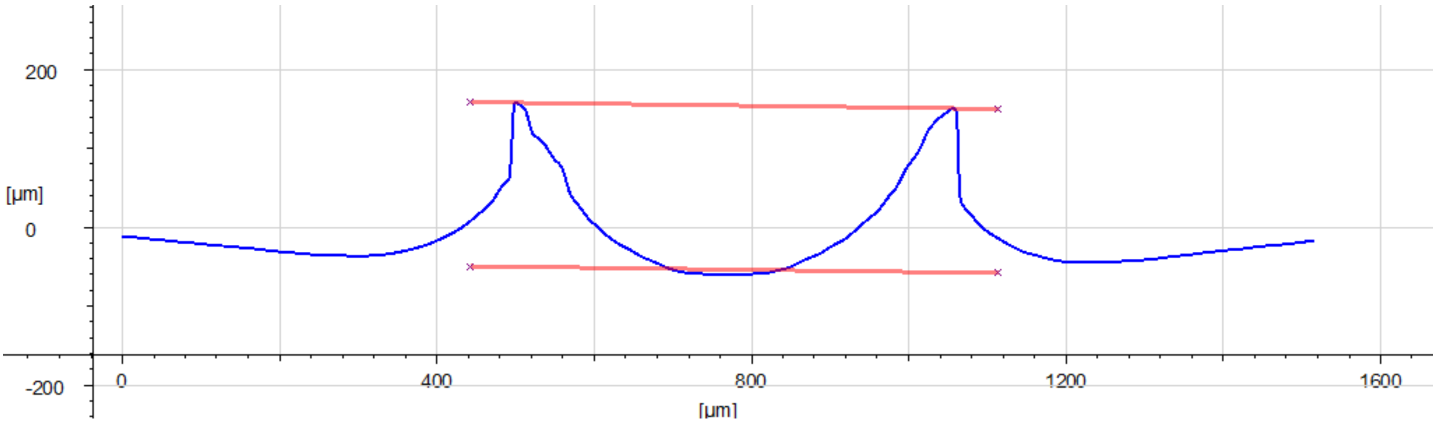

ReferencePosition	l: 1.5148mm	z: -17.5592µm
MeasurePosition	l: 0.0000nm	z: -12.4244µm
RelativeMeasurement	Δl: -1.5148mm	Δz: 5.1348µm
	Angle: 179.8058°	Distance: 1.5148mm
Filter:	No Filter - Primary profile	

Heightstep

	**Angle [°]**	**Distance [µm]**	**P1.x [µm]**	**P1.y [µm]**	**P2.x [mm]**	**P2.y [µm]**	**P3.x [µm]**	**P3.y [µm]**	**P4.x [mm]**	**P4.y [µm]**
Heightstep 1	179.2554	208.9919	442.3008	159.4001	1.1148	150.6602	442.3008	-49.6094	1.1148	-58.3493

Alicona Imaging GmbH
Dr.-Auner Strasse 21a
A-8074 Raaba/Graz

Measurement Report

Profilometry Baton 2 Sample 2A 10-8-2019 AsSc Crop

Metric size: 1.7491mm x 3.9075mm
Size: 1590 x 3552 points
2019-10-08T15:10:03

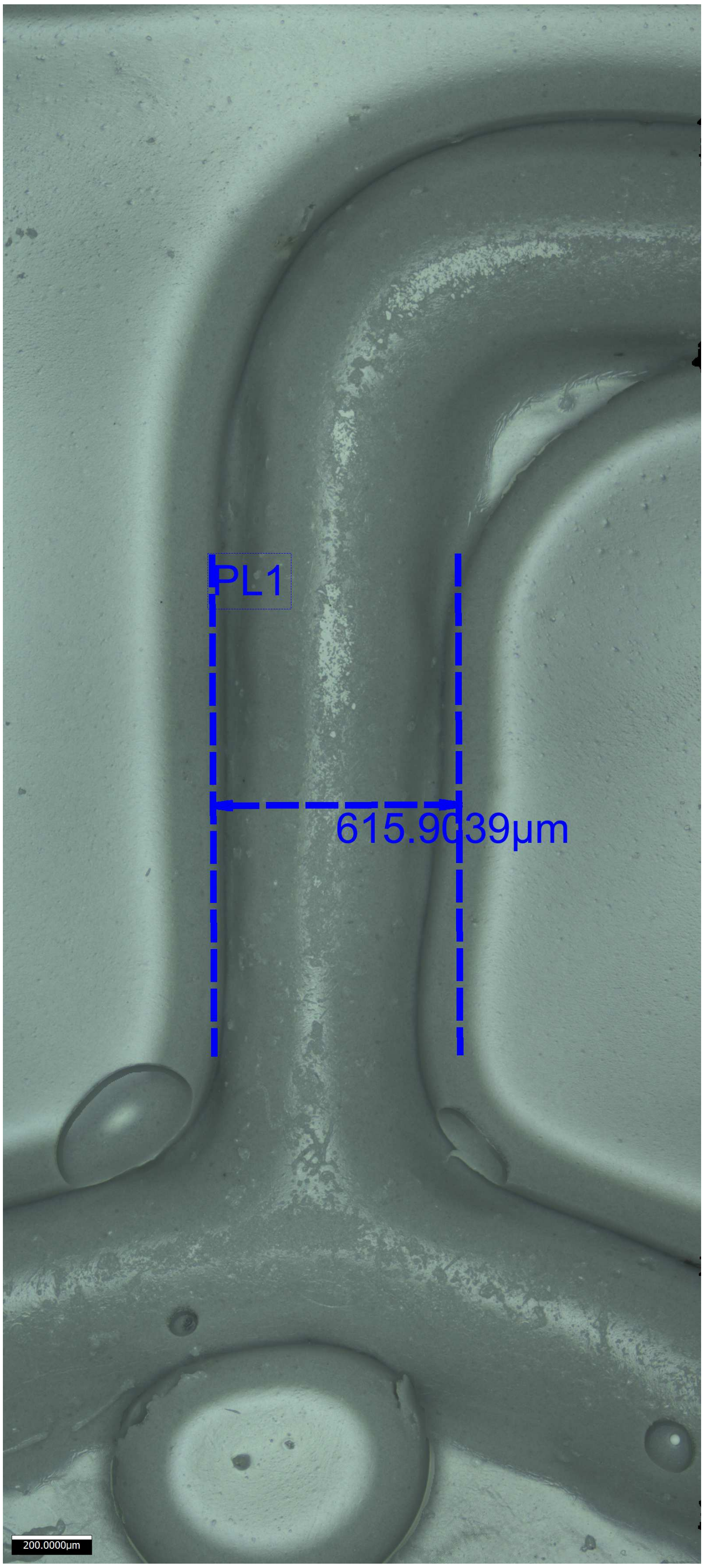

Alicona Imaging GmbH
Dr.-Auner Strasse 21a
A-8074 Raaba/Graz

Measurement Report

ProfileFormMeasurement

Measurement

Profilometry Baton 2 Sample 2A 10-8-2019 AsSc Crop

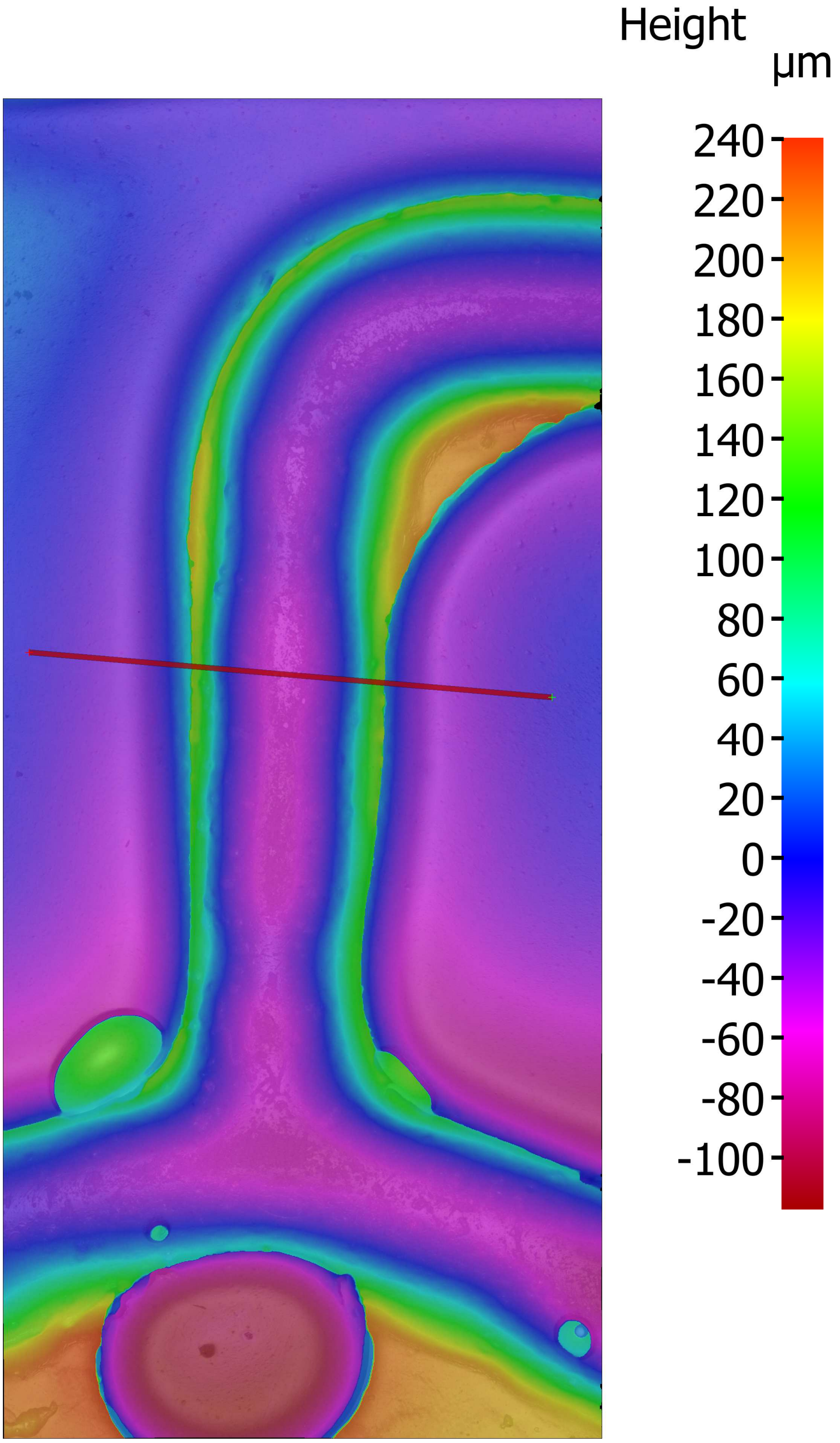

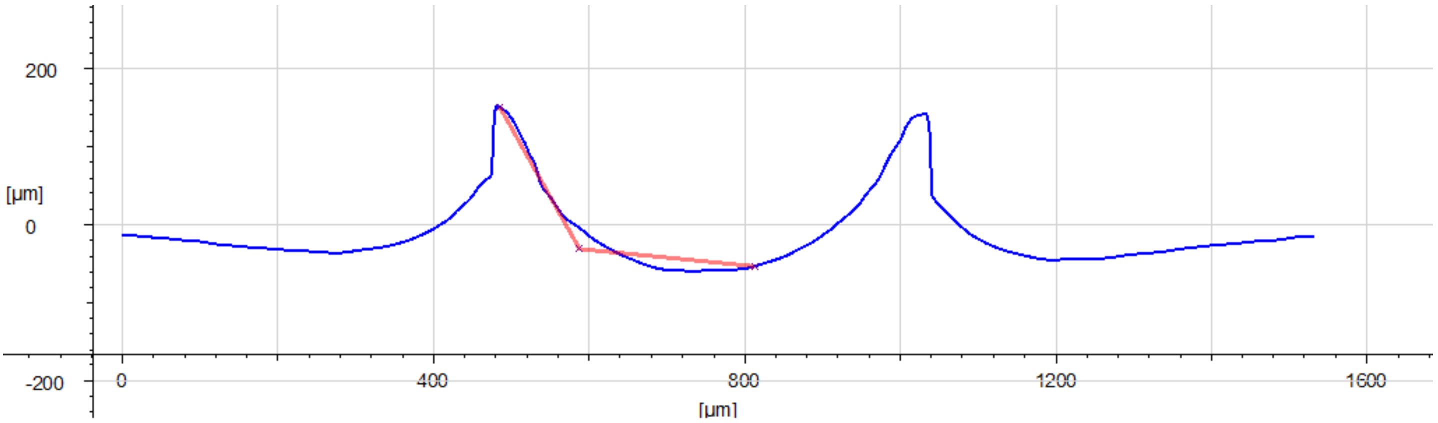

ReferencePosition	l: 1.5313mm	z: -14.6896µm
MeasurePosition	l: 0.0000nm	z: -13.6120µm
RelativeMeasurement	Δl: -1.5313mm	Δz: 1.0776µm
	Angle: 179.9597°	Distance: 1.5313mm
Filter:	No Filter - Primary profile	

Angle

	**Angle [°]**	**Apex X [µm]**	**Apex Y [µm]**
Angle 1	124.9927	587.2915	-31.3579
