# Supplementary material for: 3D printing direct to industrial roll-to-roll casting for fast prototyping of scalable microfluidic systems
Source: PLoS One. 2020 Dec 28;15(12):e0244324. doi: 10.1371/journal.pone.0244324 (PMC7769481; doi:10.1371/journal.pone.0244324)
Supplement: S3 File — (PDF) [file pone.0244324.s003.pdf]

Segment	Replicate 1	Replicate 2	Replicate 3	Replicate 4	Replicate 5	AMI	STDEV
	**PDMS**						
1	0.522747	0.525614	0.526696	0.542553	0.518327	0.527188	0.009179
2	0.504635	0.509248	0.494047	0.520341	0.507869	0.507228	0.009446
3	0.49949	0.500839	0.505962	0.534809	0.510941	0.510408	0.014372
4	0.463508	0.492987	0.493393	0.499724	0.476353	0.485193	0.014896
5	0.442899	0.464017	0.476118	0.489099	0.467647	0.467956	0.017008
6	0.390478	0.42635	0.429929	0.457933	0.42495	0.425928	0.02397
	**FILM**						
1	0.467984	0.526857	0.510005	0.518847	0.504349	0.505608	0.022712
2	0.445589	0.506934	0.509578	0.475596	0.495452	0.48663	0.026565
3	0.434438	0.477801	0.494852	0.435533	0.513547	0.471234	0.035426
4	0.455885	0.521226	0.489587	0.432404	0.478292	0.475479	0.033686
5	0.465412	0.500764	0.466923	0.426435	0.435305	0.458968	0.029453
6	0.45039	0.512655	0.487339	0.391297	0.428019	0.45394	0.047884
